# Supplementary material for: Modulating the hierarchical fibrous assembly of Au nanoparticles with atomic precision
Source: Nat Commun. 2018 Sep 24;9:3871. doi: 10.1038/s41467-018-06395-8 (PMC6155310; doi:10.1038/s41467-018-06395-8)
Supplement: Supplementary file 1 — Supplementary Information [file 41467_2018_6395_MOESM1_ESM.pdf]

Modulating the Hierarchical Fibrous Assembly of Au Nanoparticles with Atomic Precision

Li *et al*

Supplementary Methods

X-ray Crystallographic Determination of $[Au_{21}(SR)_{12}(P\text{-}C\text{-}P)_2]^+[Cl]^-$. Single crystal X-ray diffraction data of $[Au_{21}(SR)_{12}(P\text{-}C\text{-}P)_2]^+[Cl]^-$ was collected on a Bruker X8 Prospector Ultra equipped with an Apex II[1] CCD detector and an IμS micro-focus CuKα X-ray source (λ = 1.54178 Å). A piece of dark brown block crystal with dimensions 0.18 x 0.12 x 0.04 mm was mounted onto a MiTeGen MicroMount. Data collection was performed at 230.0 K under N_2 flow. A based-centered monoclinic unit cell with a = 21.5610(4) Å, b = 23.6201(4) Å, c = 28.3774(5) Å, α = 90˚, β = 94.1050(10)º, γ = 90º was determined using the least-square refinement of 9989 reflections in the range of 2.78 < θ < 59.04. The frames were integrated with the Bruker SAINT software package to yield 49640 reflections in the range of 2.78˚ < θ < 59.06˚, of which 10371 were independent (average redundancy 4.79, completeness = 100%, R_{int} = 6.03 %, R_{sigma} = 4.85 %) Data were corrected for absorption effects using SADABS Multi-Scan method (Tmax = 0.753, Tmin = 0.300). The structure was solved by intrinsic phasing using Bruker program SHELXT[2] and refined using Olex2 software in C2/c group. All the Au, Ag, S, Cl and P atoms were found directly. Remaining non-hydrogen atoms were generated via subsequent difference Fourier syntheses. All nonhydrogen atoms were refined anisotropically. Idealized hydrogen atom positions were calculated.

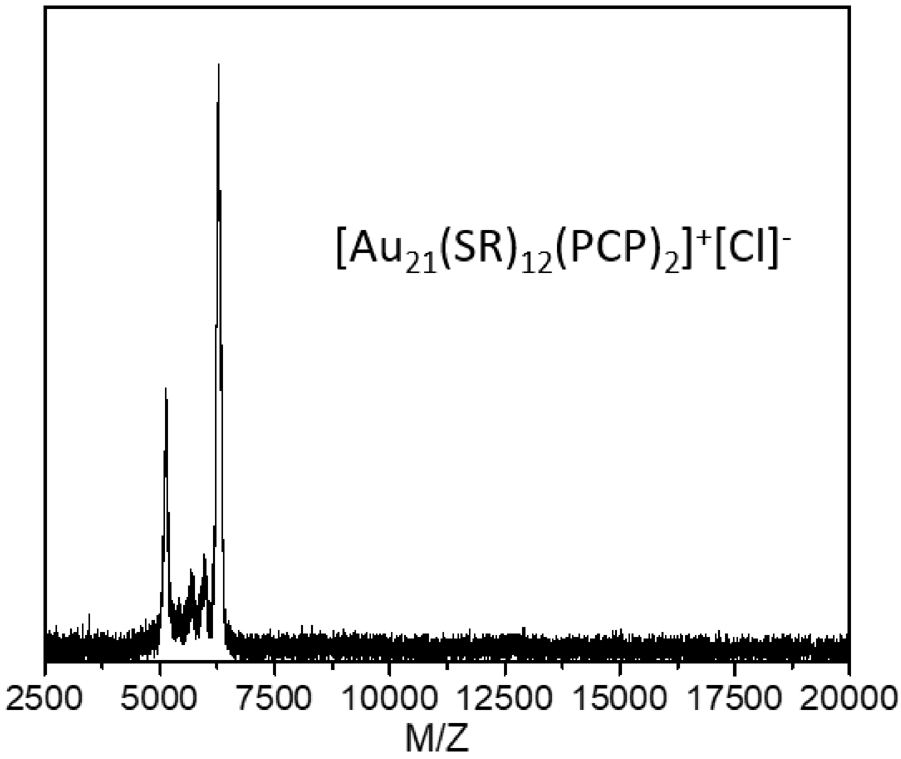

Supplementary Figure 1. Matrix-assisted laser desorption/ionization mass spectrum (MALDI-MS) of the $[Au_{21}(SR)_{12}(P\text{-}C\text{-}P)_2]^{+}[Cl]^{-}$. An intense peak at ~6285 Da is assigned to the intact $[Au_{21}(SR)_{12}(P\text{-}C\text{-}P)_2]^{+}$ (theoretical value: 6288.25 Da). Note: The lower mass peaks are fragments caused by the laser in MALDI-MS analysis. The intensities of fragments show a laser power dependence and the spacing of fragments and intact nanocluster is $(AuSR)_n$ (n: 1-4). The measurement was done in positive mode.

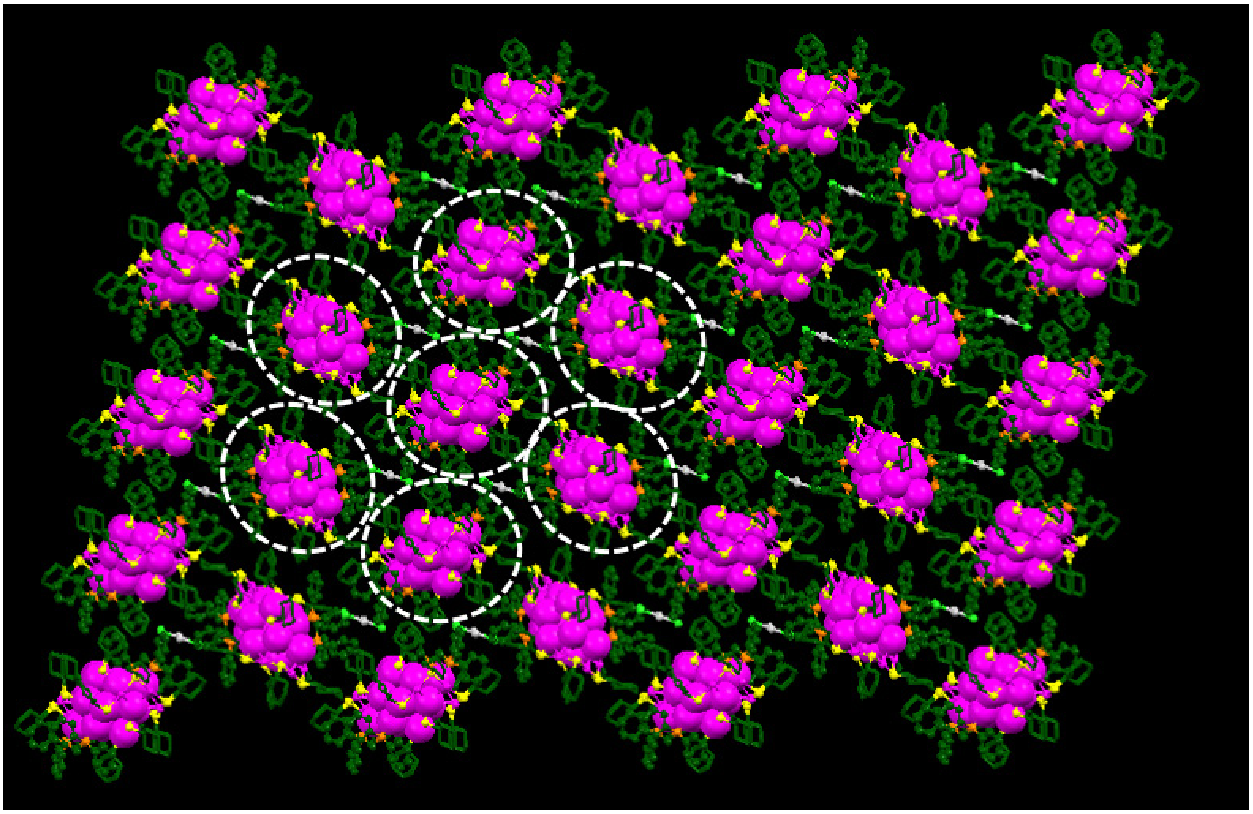

Supplementary Figure 2. The *quasi* close-packing mode (white circled) in the {100} plane in the $[Au_{21}(SR)_{12}(PCP)_2]^+[AgCl_2]^-$ single crystal.

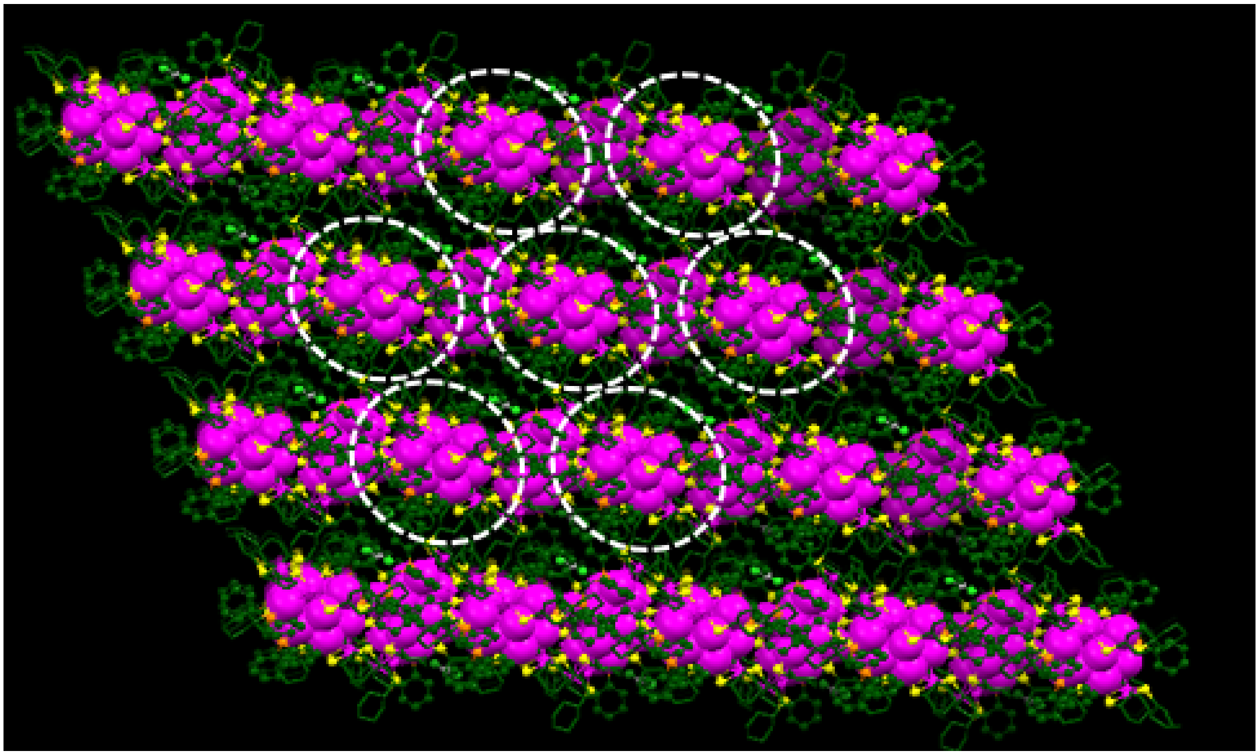

Supplementary Figure 3. The *quasi* close-packing mode (white circled) in the {001} plane in the $[Au_{21}(SR)_{12}(PCP)_2]^+[AgCl_2]^-$ single crystal.

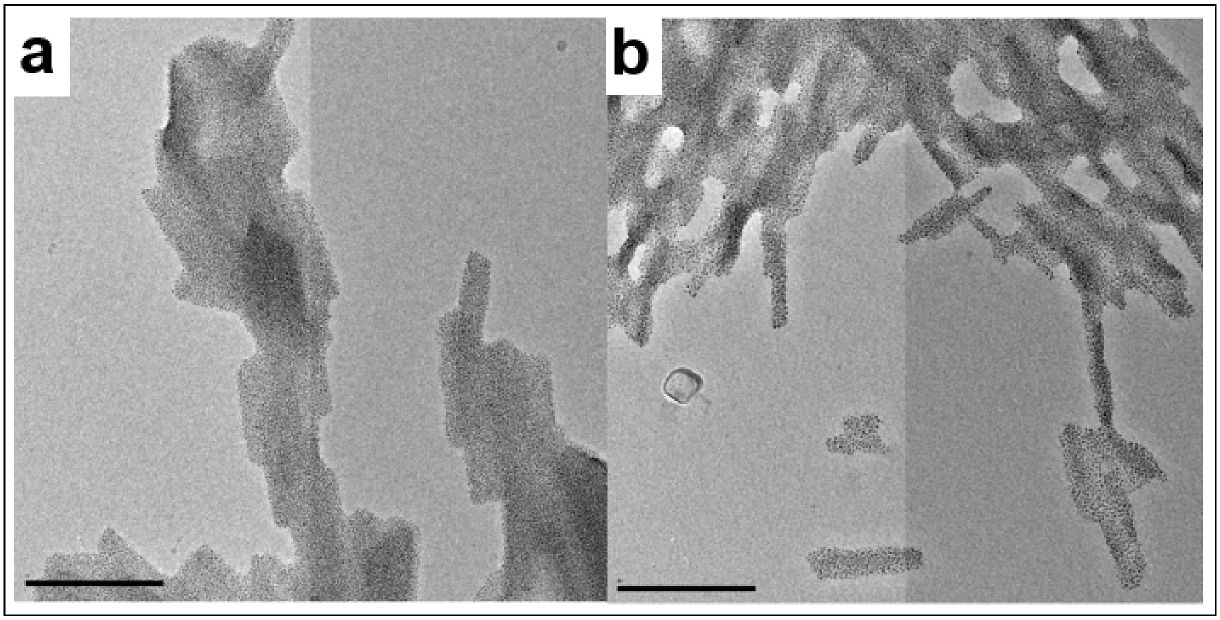

Supplementary Figure 4. The 2D nanosheets assembled from $[Au_{23}(SR)_{16}]^{-}[TOA]^{+}$ under DCM/Pentane = 1:1. (a) TEM image of one area of the sample. (b) TEM image of another area. The scale bar is 200 nm (for both panels a and b).

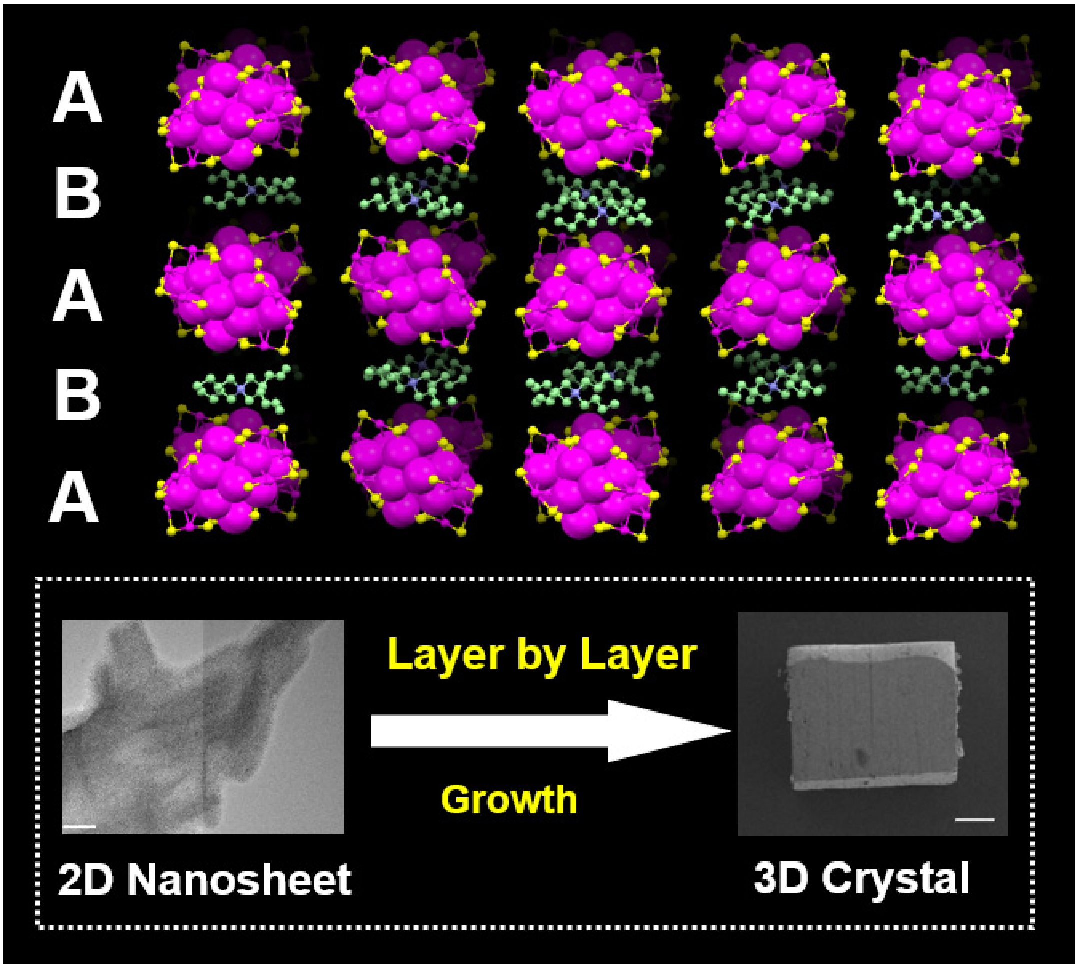

Supplementary Figure 5. The "2D to 3D" hierarchical assembly of $[Au_{23}(SR)_{16}]^{-}[TOA]^{+}$, $[Au_{23}(SR)_{16}]^{-}$ in layer A and $[TOA]^{+}$ in layer B . Inset: a TEM image of the 2D nanosheet (scale bar: 100 nm) and a SEM image of the 3D crystal (scale bar: 50 μm) assembled from $[Au_{23}(SR)_{16}]^{-}[TOA]^{+}$.

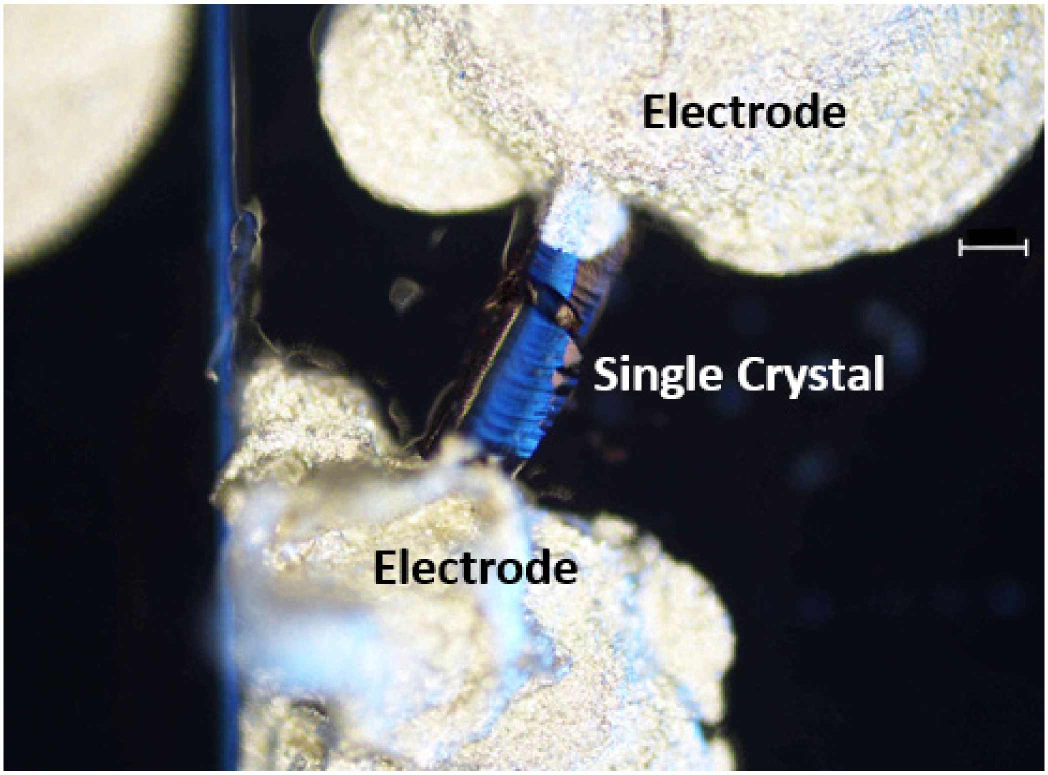

Supplementary Figure 6. Optical micrograph of a single crystal device used to measure the electrical transport properties (Scale bar: 100 μm).

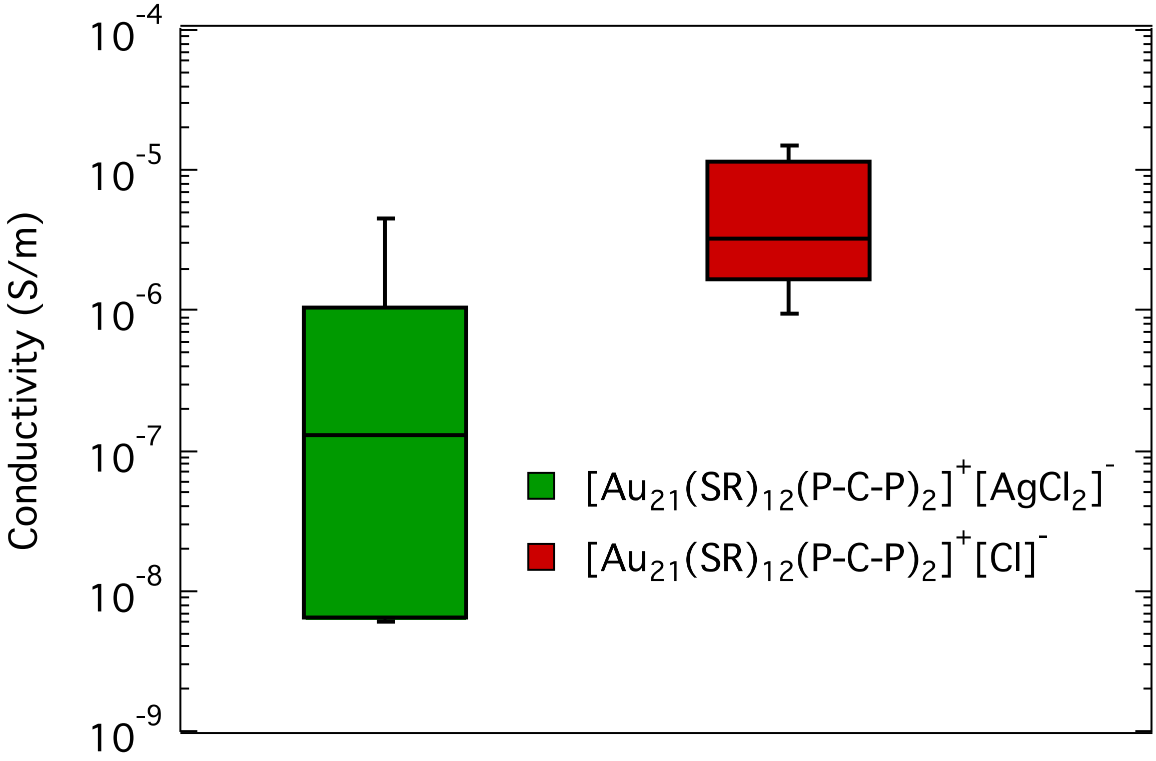

Supplementary Figure 7. Box-and-whisker plot of the set of data used to evaluate Au_{21} conductivity for two different counter-ions. Shown are median, 25 and 75% quartiles, and full range. The sample size for the case of $AgCl_2^-$ is 7 devices and for Cl^- 6 devices.

Supplementary Table 1. Crystal data and structure refinement

Identification code	ltyLQ20170123_0m_a
Empirical formula	C122H176Au21ClP4S12
Formula weight	6322.96
Temperature/K	230.01
Crystal system	monoclinic
Space group	C2/c
a/Å	21.5610(4)
b/Å	23.6201(4)
c/Å	28.3774(5)
α/°	90
β/°	94.1050(10)
γ/°	90
Volume/$Å^3$	14414.8(4)
Z	4
ρ calcg/cm^3	2.914
μ/mm^{-1}	41.551
F(000)	11344.0
Crystal size/mm^3	0.11 × 0.03 × 0.02
Radiation CuKα	(λ = 1.54178)
2Θ range for data collection/°	5.558 to 118.128
Index ranges	-23 ≤ h ≤ 23, -26 ≤ k ≤ 25, -27 ≤ l ≤ 31
Reflections collected	49640
Independent reflections	10371 [Rint = 0.0603, Rsigma = 0.0485]
Data/restraints/parameters	10371/0/723
Goodness-of-fit on F^2	1.074
Final R indexes [I>=2σ (I)]	R1 = 0.0386, wR2 = 0.0934
Final R indexes [all data]	R1 = 0.0493, wR2 = 0.0988
Largest diff. peak/hole / e $Å^{-3}$	1.83/-2.17

Supplementary References

[1]. APEX II software suite, *Bruker-AXS* (2006).

[2]. G. M. Sheldrick, *Acta Crystallogr. A*, **64**, 112 (2008).
